# Palmitoylethanolamide Treatment Reduces Blood Pressure in Spontaneously Hypertensive Rats: Involvement of Cytochrome P450-Derived Eicosanoids and Renin Angiotensin System

**DOI:** 10.1371/journal.pone.0123602

**Published:** 2015-05-07

**Authors:** Giuseppina Mattace Raso, Claudio Pirozzi, Roberta d'Emmanuele di Villa Bianca, Raffaele Simeoli, Anna Santoro, Adriano Lama, Francesca Di Guida, Roberto Russo, Carmen De Caro, Raffaella Sorrentino, Antonio Calignano, Rosaria Meli

**Affiliations:** 1 Department of Pharmacy, University of Naples Federico II, Naples, Italy; 2 The William Harvey Research Institute, Barts and The London School of Medicine, Queen Mary University of London, London, United Kingdom; University of Calgary, CANADA

## Abstract

Palmitoylethanolamide (PEA), a peroxisome proliferator-activated receptor-α agonist, has been demonstrated to reduce blood pressure and kidney damage secondary to hypertension in spontaneously hypertensive rat (SHR). Currently, no information is available concerning the putative effect of PEA on modulating vascular tone. Here, we investigate the mechanisms underpinning PEA blood pressure lowering effect, exploring the contribution of epoxyeicosatrienoic acids, CYP-dependent arachidonic acid metabolites, as endothelium-derived hyperpolarizing factors (EDHF), and renin angiotensin system (RAS) modulation. To achieve this aim SHR and Wistar-Kyoto rats were treated with PEA (30 mg/kg/day) for five weeks. Functional evaluations on mesenteric bed were performed to analyze EDHF-mediated vasodilation. Moreover, mesenteric bed and carotid were harvested to measure CYP2C23 and CYP2J2, the key isoenzymes in the formation of epoxyeicosatrienoic acids, and the soluble epoxide hydrolase, which is responsible for their degradation in the corresponding diols. Effect of PEA on RAS modulation was investigated by analyzing angiotensin converting enzyme and angiotensin receptor 1 expression. Here, we showed that EDHF-mediated dilation in response to acetylcholine was increased in mesenteric beds of PEA-treated SHR. Western blot analysis revealed that the increase in CYP2C23 and CYP2J2 observed in SHR was significantly attenuated in mesenteric beds of PEA-treated SHR, but unchanged in the carotids. Interestingly, in both vascular tissues, PEA significantly decreased the soluble epoxide hydrolase protein level, accompanied by a reduced serum concentration of its metabolite 14-15 dihydroxyeicosatrienoic acid, implying a reduction in epoxyeicosatrienoic acid hydrolisis. Moreover, PEA treatment down-regulated angiotensin receptor 1 and angiotensin converting enzyme expression, indicating a reduction in angiotensin II-mediated effects. Consistently, a damping of the activation of angiotensin receptor 1 underlying pathways in mesenteric beds was shown in basal conditions in PEA-treated SHR. In conclusion, our data demonstrate the involvement of epoxyeicosatrienoic acids and renin angiotensin system in the blood pressure lowering effect of PEA.

## Introduction

The endothelium plays an important role in maintaining vascular homeostasis by synthesizing and releasing a spectrum of vasoactive substances [1]. Among the released vasodilating factors, prostacyclin, nitric oxide (NO) and a family of endothelium-derived hyperpolarizing factors (EDHFs) are the main actors. Endothelium-derived NO mediates vascular relaxation of relatively large, conduit arteries (i.e., aorta and epicardial coronary arteries), while EDHF plays an important role in modulating vascular tone in small resistance arteries in rodents [2–4] and in human forearm microcirculation [5,6]. Although the nature of EDHF has not been fully elucidated, different EDHFs could exist depending on species, blood vessels, and the size of blood vessels tested [7,8]. Epoxyeicosatrienoic acid (EET) pathway seems to be one of the most convincing candidate: in fact, several evidence indicate that EETs act as EDHFs in arteries from a variety of species, including humans [9]. EETs are cythocrome P450 (CYP) epoxygenase metabolites of arachidonic acid, produced by the vascular endothelium in response to agonists, such as bradykinin and acetylcholine (Ach), or physical stimulus, such as shear stress [10]. EETs are recognized as major regulators of renal and vascular functions, including vasodilation, inflammation, diuresis, and tubular fluid-electrolyte transport actions, that are predictive of a hypotensive effect. Among the metabolic pathways of arachidonic acid, CYP2C and CYP2J are the major isoforms, leading to 5,6-, 8,9-, 11,12-, or 14,15-EET regioisomers, even if 11,12- and 14,15-EETs are the predominant metabolites, believed to be the EDHFs responsible of dilation of vascular beds [11]. EETs are hydrolyzed by soluble epoxide hydrolase (sEH) in the corresponding inactive diols, dihydroxyeicosatrienoic acids (DHETs), resulting in attenuation of the vasodilation and anti-inflammatory effect of EETs. Recent studies on rat models have shown a positive correlation between sEH expression, angiotensin (Ang) II, and the elevation of blood pressure. In addition, there is accumulating evidence that stimulation of the angiotensin receptor (AT)1 participates in vascular dysfunction by reducing activity of the endothelium-derived relaxants factors, such as EDHFs [12]. EET hydrolysis has been found to be increased in renal fractions of spontaneously hypertensive rat (SHR), an animal model of Ang II-mediated hypertension [13]. Consistently, sEH was found increased in SHR renal microsomes and cytosol [13] and in renal microvessels of Ang II-induced hypertensive rats [14]. sEH expression has been shown to be also increased in aortas from saline-fed SHR or Ang II-treated normotensive rats. The transcriptional regulation of sEH expression by Ang II has been demonstrated to be mediated by AT1, since a selective AT1 antagonist reversed this effect [15]. Therefore, the increased expression of sEH has been interpreted as a result of AT1 and downstream signaling cascade activation, leading to activator protein (AP)-1 transcriptional activity. The enhanced hydrolysis of EETs in DHETs by sEH would unbalance vascular tone and hence increase systemic blood pressure.

In this scenario, it has been emerging the involvement of peroxisome proliferators-activated receptor (PPAR)α in the control of renal vascular tone [16]. For instance, the chronic fenofibrate treatment in obese Zucker rats improved the endothelium function increasing CYP-derived eicosanoid synthesis in kidney [16]. Moreover, we recently demonstrated that palmitoylethanolamide (PEA), an endogenous PPARα agonist, protects the kidney from the hypertensive injury, through the increase in the antioxidant defense [17].

This study focused particularly on mesenteric bed and carotid artery modifications to examine the mechanisms underpinning PEA lowering effect on systolic blood pressure and thus on vascular tone in SHR strain. To this aim, functional data evaluating EDHF contribution after PEA treatment have been addressed by using mesenteric bed. Moreover, to address EET role, we evaluated the modulation of CYP enzymes and sEH expression in the vasculature, together with serum DHET level. Finally, because of the close association of the Ang II/sEH/EET system and blood pressure regulation, the modulation of renin angiotensin system (RAS) by PEA has been evaluated, through measuring AT1 and angiotensin converting enzyme (ACE) expression.

## Materials and Methods

### Ethics statement

This study was carried out in strict accordance with the Institutional Guidelines and complied with the Italian D.L. no.116 of January 27, 1992 of Ministero della Salute and associated guidelines in the European Communities Council Directive of November 24, 1986 (86/609/ECC). All animal procedures reported herein were approved by the Institutional Animal Care and Use Committee (CSV) of University of Naples “Federico II” under protocol no. 2012–0081829.

### Animals and treatment

Eight-week-old male SHRs and age-matched Wistar Kyoto normotensive (WKY) rats, obtained from Harlan Italy (San Pietro al Natisone, Udine, Italy), were used for this study. Animals were housed in temperature (23±2°C)- and light-controlled (12:12-h light-dark cycle) condition and food and water freely available. The animals were divided into four groups (n = 10 each): (1) WKY and (2) SHR control; (3) WKY and (4) SHR given PEA at a dose of 30 mg kg^-1^ d^-1^ subcutaneously. PEA (Tocris Cookson Ltd., UK) was dissolved in PEG400 and Tween 80 2:1 (Sigma-Aldrich, Milan, Italy), and kept overnight under gentle agitation with a micro stirring bar. Before injection, sterile saline was added so that the final concentrations of PEG400 and Tween 80 were 20 and 10% v/v, respectively. Control WKY and SHR received vehicle. All animals were treated for 35 days (five weeks). Throughout the experimental period, systolic blood pressure (SBP) and heart rate (HR) were monitored in conscious rats. At the end of the treatment period, animals were manipulated by using different protocols for biochemical determinations or functional studies as reported below. Prior to sample collection, animals were anesthetized and euthanized to minimize pain. All efforts were made to minimize animal suffering.

### Measurement of arterial blood pressure and heart rate in conscious rats

SBP and HR were measured in conscious rats with a non-invasive common indirect method using a tail-cuff device in combination with blood flow sensor and recorder (Ugo Basile, Biological Research Apparatus, 21025 Comerio, Italy) [18]. Briefly, rats were housed for 30 min in a warmed room (28–30°C), then a tail cuff was placed about 2 cm from the base of the tail for measuring systolic blood pressure. Care was taken in selecting an appropriate cuff size for each animal. Rats were allowed to habituate to this procedure for 2 weeks before experiments were performed. Heart rate was detected by a pulse rate counter placed distal to the tail cuff and monitored with the audio signal. SBP and HR were measured between 09.00h and 12.00h and values were recorded and were averaged from at least three consecutive readings per rat.

### 14,15-DHET serum measurement

At the end of the treatment period, animals were anesthetized by enflurane and before vessel withdraw for Western blot analysis, blood was collected for serum determination of 14,15-DHET through an enzyme-linked immunosorbent assay (14,15-DHET ELISA kit; Detroit R&D Inc., Detroit, MI, USA), according to the manufacturer's instructions.

### Isolated and perfused mesenteric bed

Mesenteric bed preparation was performed as previously reported by d’Emmanuele di Villa Bianca [19]. In brief, rats were euthanized and the superior mesenteric artery was cannulated to perfuse the whole vascular bed with Krebs’ buffer containing heparin (10 IU/ml; Sigma-Aldrich, Milan, Italy) for 5 min at 2 ml/min. In order to measure changes in perfusion pressure, the mesenteric bed was separated from the intestine by cutting along the closed intestinal border and connected to a pressure transducer (Bentley 800 Trantec; Ugo Basile, Comerio, Italy). It was perfused with Krebs’ buffer (2 ml/min) composed of 115.3 mM NaCl, 4.9 mM KCl, 1.46 mM CaCl_2_, 1.2 mM MgSO_4_, 1.2 mM KH_2_PO_4_, 25.0 mM NaHCO_3_, and 11.1 mM glucose (Carlo Erba Reagents, Milan, Italy), warmed at 37°C, and oxygenated (95% O_2_, 5% CO_2_). After approximately 20 min of equilibration, methoxamine (MTX, 100 μM; Sigma-Aldrich), an adrenergic α_1_-agonist was added to the Krebs’ solution. In order, to visualize NO synthase (NOS) and cyclooxygenase (COX)-independent relaxation i.e., EDHF, a concentration-response curve of acetylcholine (Ach bolus injection; 1-10-100-1000 pmoles; Sigma-Aldrich; Milan, Italy) was performed on stable tone of MTX in Krebs’ solution medicated with indomethacin (INDO; 10μM; Sigma-Aldrich) and N^G^-nitro-L-arginine methyl ester (L-NAME, 100μM; Sigma-Aldrich) as COX and NOS inhibitors, respectively. The increase in perfusion pressure, i.e. contraction, was expressed as mmHg. The decrease in perfusion pressure, i.e. EDHF-mediated vasodilation, was calculated as area under the curve (mmHg x s) to visualize the effect throughout the time.

### Western blot analysis

Mesenteric tissues and carotids were excised, harvested, frozen in liquid nitrogen and stored for protein evaluations. Later tissues were homogenized on ice in lysis buffer (Tris-HCl, 20 mM pH 7.5, 10 mM NaF, 150 mM NaCl, 1% Nonidet P-40, 1 mM phenylmethylsulphonyl fluoride, 1 mM Na_3_VO_4_, 10 μg/ml leupeptin and trypsin inhibitor). After 1 h, tissue lysates were obtained by centrifugation at 13000 rpm for 20 min at 4°C. The protein concentration of the samples was determined by Bio-Rad protein assay (Bio-Rad Laboratories, Segrate, Milan, Italy), using bovine serum albumin as standard.

For Western blot analysis, 35 μg protein of tissue lysate was dissolved in Laemmli’s sample buffer, boiled for 5 min, and subjected to SDS-PAGE (8% or 12% polyacrylamide). The blot was performed by transferring proteins from a slab gel to nitrocellulose membrane at 240 mA for 40 min at room temperature. The filter was then blocked with 1x PBS, 5% non fat dried milk for 40 min at room temperature and probed with rabbit polyclonal antibodies anti-CYP2C23 (1:500, kindly provided by Prof. Jorge H. Capdevila, Vanderbilt, University Medical School, Nashville, TN) or anti- sEH or anti-AT-1 or anti-ACE (1:2000; Upstate Biotechnology, Lake Placid, NY, USA), anti-CYP2J2 (1:500, kindly provided by Dr. Darryl C. Zeldin, National Institute of Environmental Health Science, Research Triangle Park, NC) or anti-IκBα (1:200, Santa Cruz Biotechnology, Inc., Santa Cruz, CA, USA), or phospho-signal transducer and activator of transcription (STAT)3 or STAT3 (1:1000; Cell Signaling Technology, Danvers, MA, USA) or phospho-extracellular signal-regulated-kinases (ERK) 1/2 (1:200; Cell Signaling Technology, Danvers, MA, USA) dissolved in 1x PBS, 5% non fat dried milk, 0.1% Tween 20 at room temperature, overnight or for 2h. The secondary antibody (anti-rabbit IgG-horseradish peroxidase conjugate 1:5000 dilution) was incubated for 1 h at room temperature. Subsequently, the blot was extensively washed with PBS, developed using enhanced chemiluminescence detection reagents (Amersham Pharmacia Biotech, Piscataway, NJ, Piscataway, NJ, USA) according to the manufacturer’s instructions and the immune complex visualized by Imag Quant.

The protein bands were scanned and densitometrically analyzed with a model GS-700 imaging densitometer (Bio-Rad Laboratories, Milan, Italy). Western blot for α-tubulin (Sigma; St. Louis, MO, USA) was performed to ensure equal sample loading.

### Statistical analysis

All data were presented as mean ± S.E.M. All analysis was conducted using Graph-Pad Prism (Graph-Pad software Inc., San Diego, CA, USA). Statistical analysis was performed by ANOVA test for multiple comparisons followed by Bonferroni’s test. Statistical significance was set at P <0.05.

## Results

### Effect of PEA on blood pressure and heart rate

SHR rats had a significant increase in SBP (mmHg) values compared to WKY rats (220.2±11.1 vs 156.8±3.3; P<0.001). No significant differences in blood pressure were observed in PEA-treated WKY rats (125.4±8.4) compared to WKY, even if a not significant trend of decrease was shown. Conversely, a lowering effect on blood pressure was shown following PEA treatment of SHR for 5 weeks (171.0±8.8) compared to untreated SHR (P<0.01). No changes were observed in HR following PEA treatment both of WKY and SHR (data not shown).

### EDHF involvement in mesenteric arterial bed

**Fig 1:**
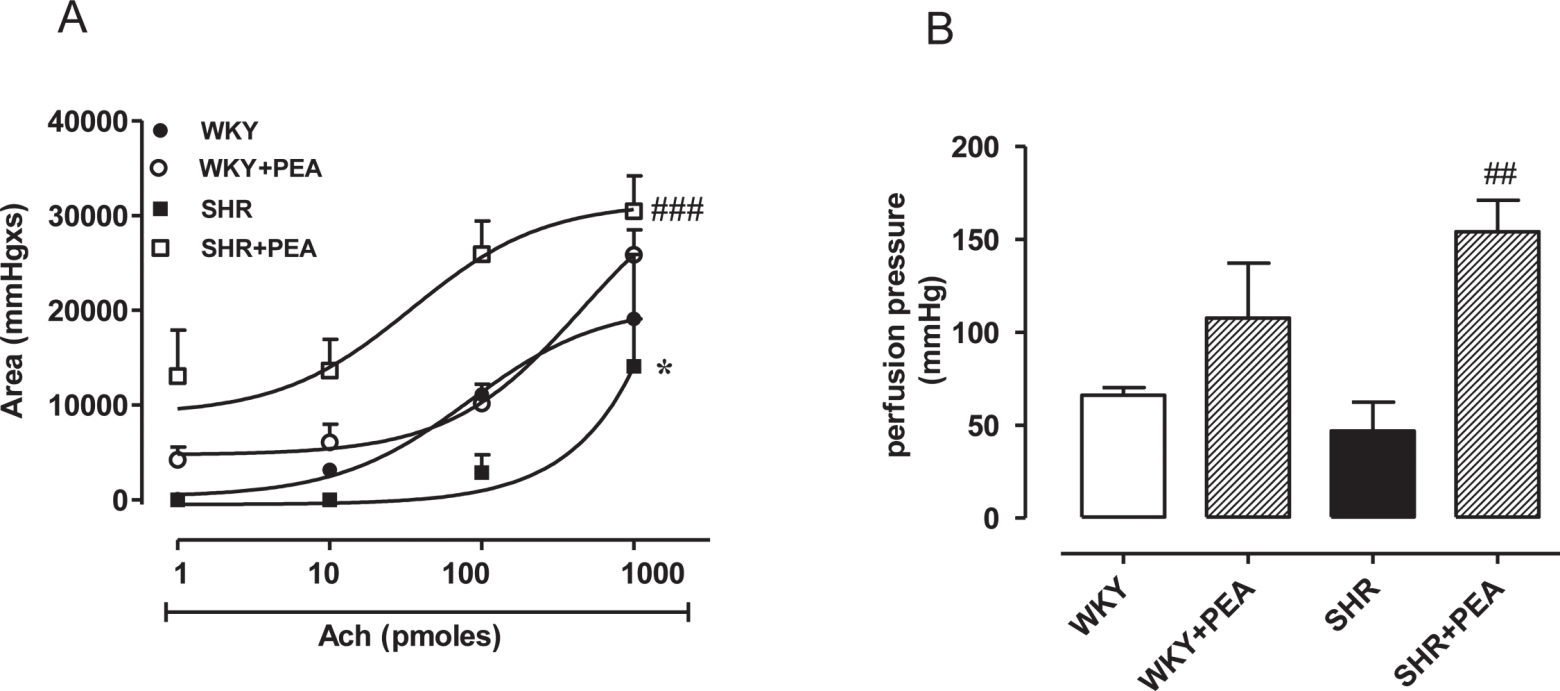
Effect of PEA on EDHF-mediated relaxation on mesenteric arterial bed on stable tone of MTX. In order to visualize the contribution of EDHF in mesenteric bed, a curve concentration-response to Ach (1–1000 pmoles) in Krebs solution medicated with INDO (10 μM) and L-NAME (100 μM) was performed on MTX stable tone. (A) The EDHF-mediated relaxation resulted significantly reduced in SHR compared with WKY (*P<0.05). The treatment with PEA significantly increased EDHF-mediated relaxation in SHR compared with SHR group (###P<0.001). (B) The increase in perfusion pressure (mmHg) achieved by the adding of INDO (10 μM) plus L-NAME (100 μM) was significantly higher in PEA-treated SHR compared to SHR (##P<0.01). Data are expressed as means ± SEM for 4–5 different animals.

In order to visualize the contribution of EDHF in mesenteric bed, as NOS- and COX-independent relaxation, a concentration-response curve to Ach (1–1000 pmoles) in Krebs solution medicated with INDO (10 μM) and L-NAME (100 μM) was performed on MTX stable tone. The EDHF-mediated relaxation resulted significantly reduced in SHR compared with WKY (Fig 1A, P<0.05). Interestingly, the treatment with PEA caused a significant increase in EDHF-mediated relaxation in SHR compared with SHR untreated group (Fig 1A; P<0.001). No significant effect was observed in WKY rats after PEA treatment (Fig 1A). Similar results were also obtained calculating the increase in contraction achieved by adding INDO (10 μM) and L-NAME (100 μM) in mesenteric arterial bed from all treated groups. The increase in perfusion pressure i.e. contraction was significantly higher in SHR treated with PEA compared with SHR (P<0.001; Fig 1B). In WKY rats treatment with PEA caused an increase in contraction even if not significant. To note, the basal perfusion pressure and MTX-induced tone was comparable among all groups.

### PEA effect on vascular expression of CYP2C23 and CYP2J2

**Fig 2:**
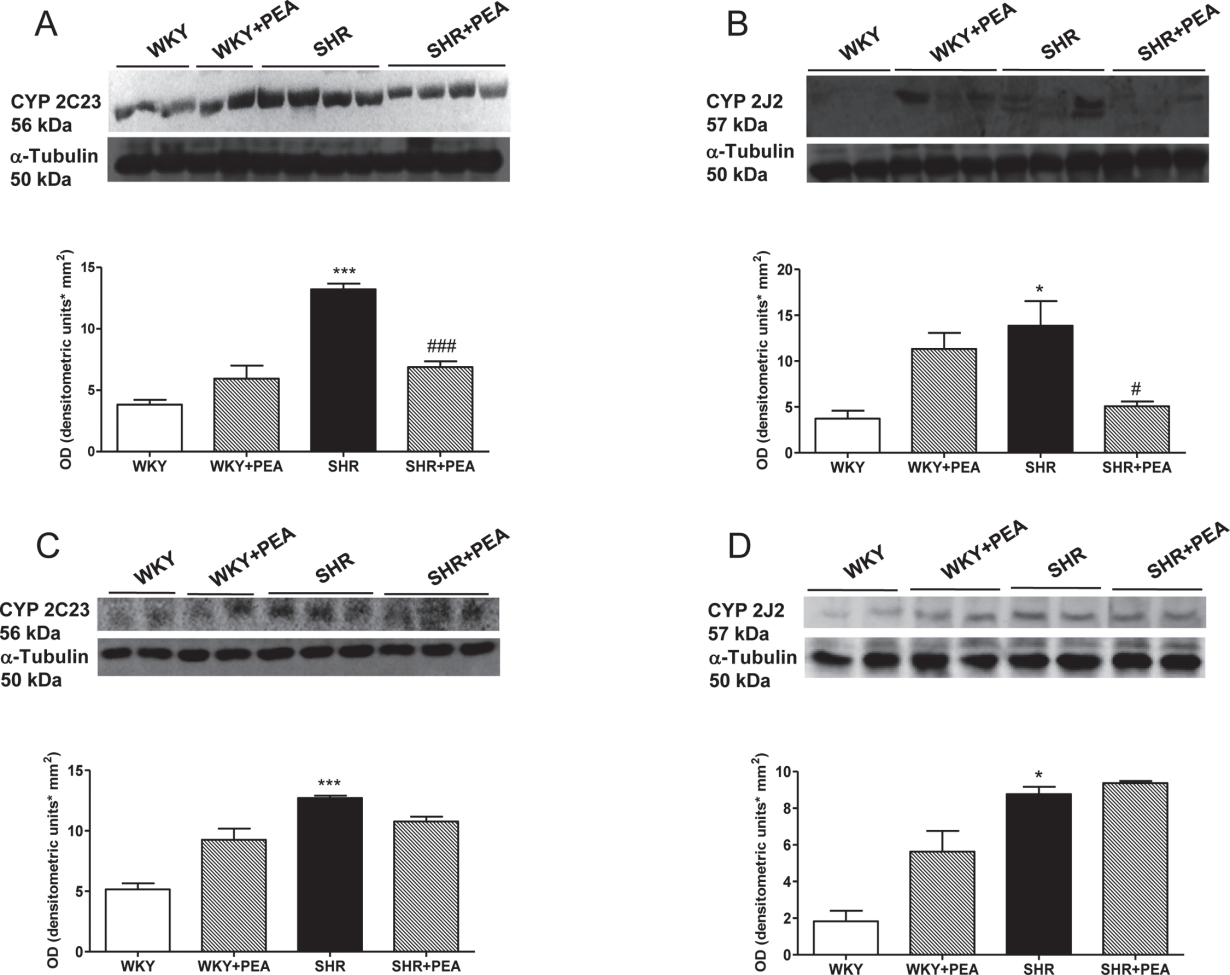
Effect of PEA on CYP2C23 and CYP2J2 protein expression in mesenteric bed and carotid. Representative Western blots show bands in mesenteric bed (A and B) and carotid (C and D) of SHR and WKY rats. Densitometric evaluations of protein levels were obtained from 5 different animals. Data are expressed as means ± SEM. *P<0.05 and ***P<0.001 vs WKY; #P<0.05 and ###P<0.001 vs SHR.

CYP enzymes constitute a major catabolic pathway for arachidonic acid to generate EETs, involved in the control of vascular tone. Indeed, CYP2J and CYP2C isoforms have been reported to catalyze the synthesis of EETs [20]. Fig 2 shows representative Western blots for CYP2C23 and CYP2J2 protein in mesenteric bed (A and B) and carotid (C and D) of WKY and SHR rats. While PEA significantly prevented the increased expression of both CYP proteins in the mesenteric bed from SHR (Fig 2A and 2B), their expression was not different between carotids from untreated and PEA-treated SHR (Fig 2C and 2D). No significant difference was observed in CYP2C23 and CYP2J2 in both vascular tissues in PEA-treated WKY rats compared to untreated WKY rats.

### PEA decreases sEH expression in the vasculature and DHETs in serum of SHR animals

**Fig 3:**
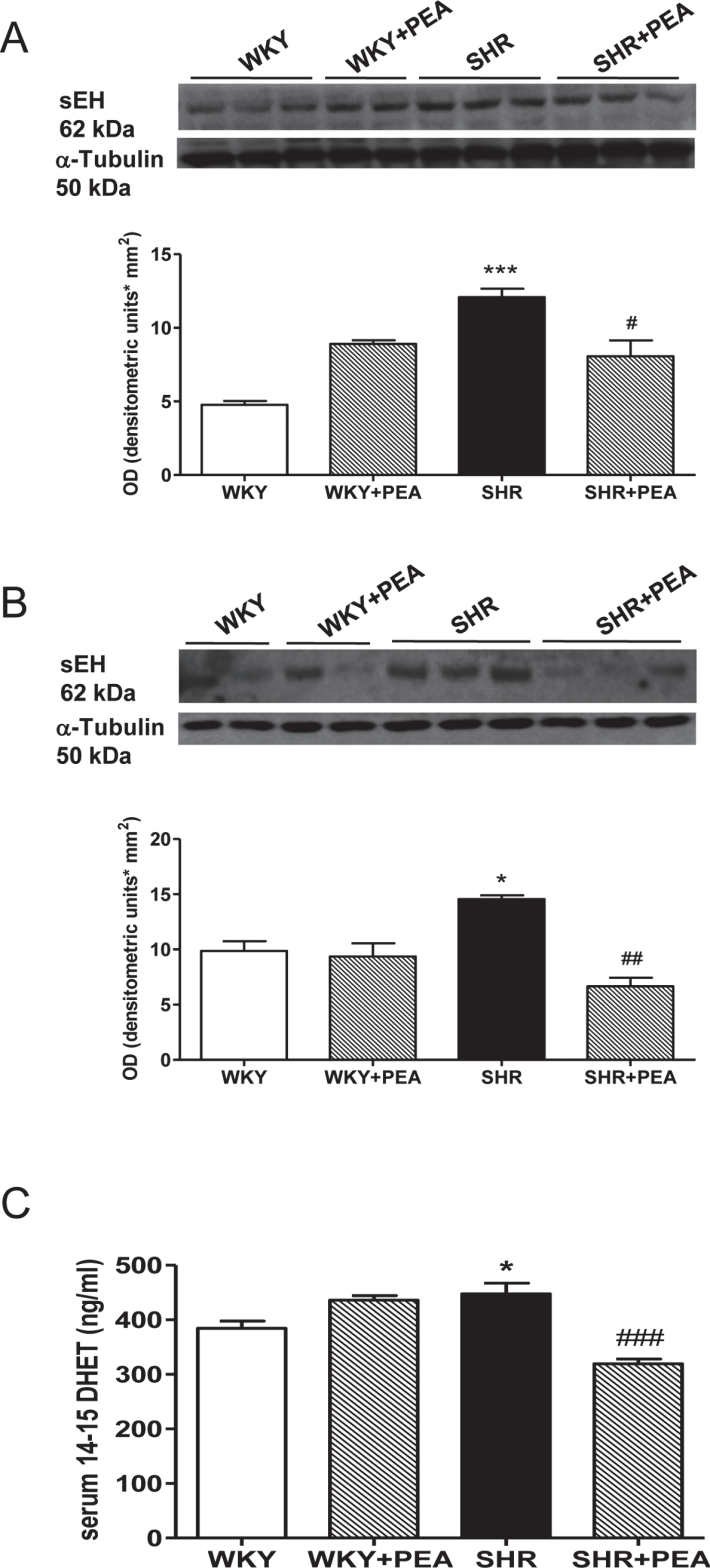
sEH protein expression in mesenteric bed and carotid and its modulation by PEA treatment. Immunoblot of sEH and densitometric analysis of protein band from mesenteric bed (A) and carotid (B) of SHR and WKY rats are shown. Serum 14-15DHET (ng/ml) is reported in panel C. Data are expressed as means ± SEM. *P<0.05 and ***P<0.001 vs WKY; #P<0.05, ##P<0.01, and and ###P<0.001 vs SHR.

To evaluate systemic EET hydrolisis, we analyzed sEH expression and EET formation, by western blot analysis of vascular tissues and DHETs concentration in serum of all animals, respectively. sEH is implicated in blood pressure control by virtue of its ability to degrade EETs that exert vasodilatory effects. Indeed, it is already known that sEH protein expression is increased in SHR [13], consistently with its hypertensive role. As shown in Fig 3, immunoblots performed on mesenteric bed (panel A) and carotid (panel B) from SHR animals revealed a marked increase in sEH expression, which was significantly prevented by PEA treatment. Consistently, the increase in serum 14,15-DHETs shown in SHR, was significantly prevented by PEA (Fig 3C), suggesting a reduced hydrolisis of EETs in PEA-treated SHR and hence an increase of their concentration.

### PEA modulation of RAS in the vasculature

**Fig 4:**
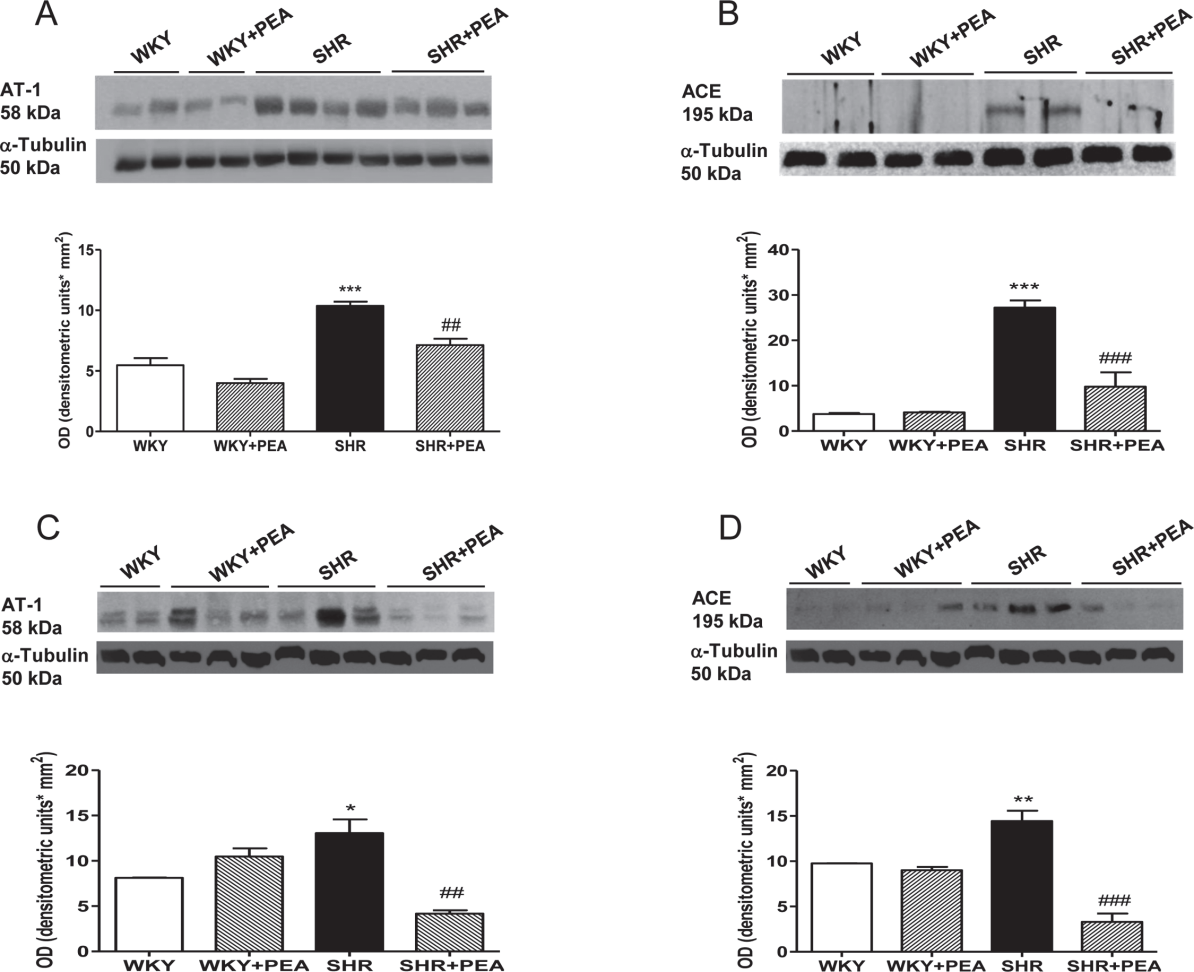
Effect of PEA on AT1 and ACE protein expression in mesenteric bed and carotid. Representative Western blots show bands in mesenteric bed (A and B) and carotid (C and D) of SHR and WKY rats. Densitometric evaluations of protein levels were obtained from 5 different animals. Data are expressed as means ± SEM. *P<0.05, **P<0.01 and ***P<0.001 vs WKY; ##P<0.01 and ###P<0.001 vs SHR.

To evaluate whether the hemodynamic effects of PEA bears any relationship to the modulation of RAS, we determined the expression of AT1 receptors and ACE in the vasculature from SHR. As shown in Fig 4, AT1 expression has been found increased both in mesenteric bed and carotid in SHR compared with WKY (panel A and C, respectively), whereas it significantly decreased in SHRs treated with PEA.

Thereafter, we also evaluated the vascular source of Ang II, determining ACE protein expression. As shown in Fig 4B and 4D, the increased expression of ACE enzyme in both vascular tissues of SHR, was blunted by PEA treatment in mesenteric bed and carotid, respectively.

### Reduction of AT1-mediated signaling pathway activation in mesenteric bed by PEA

**Fig 5:**
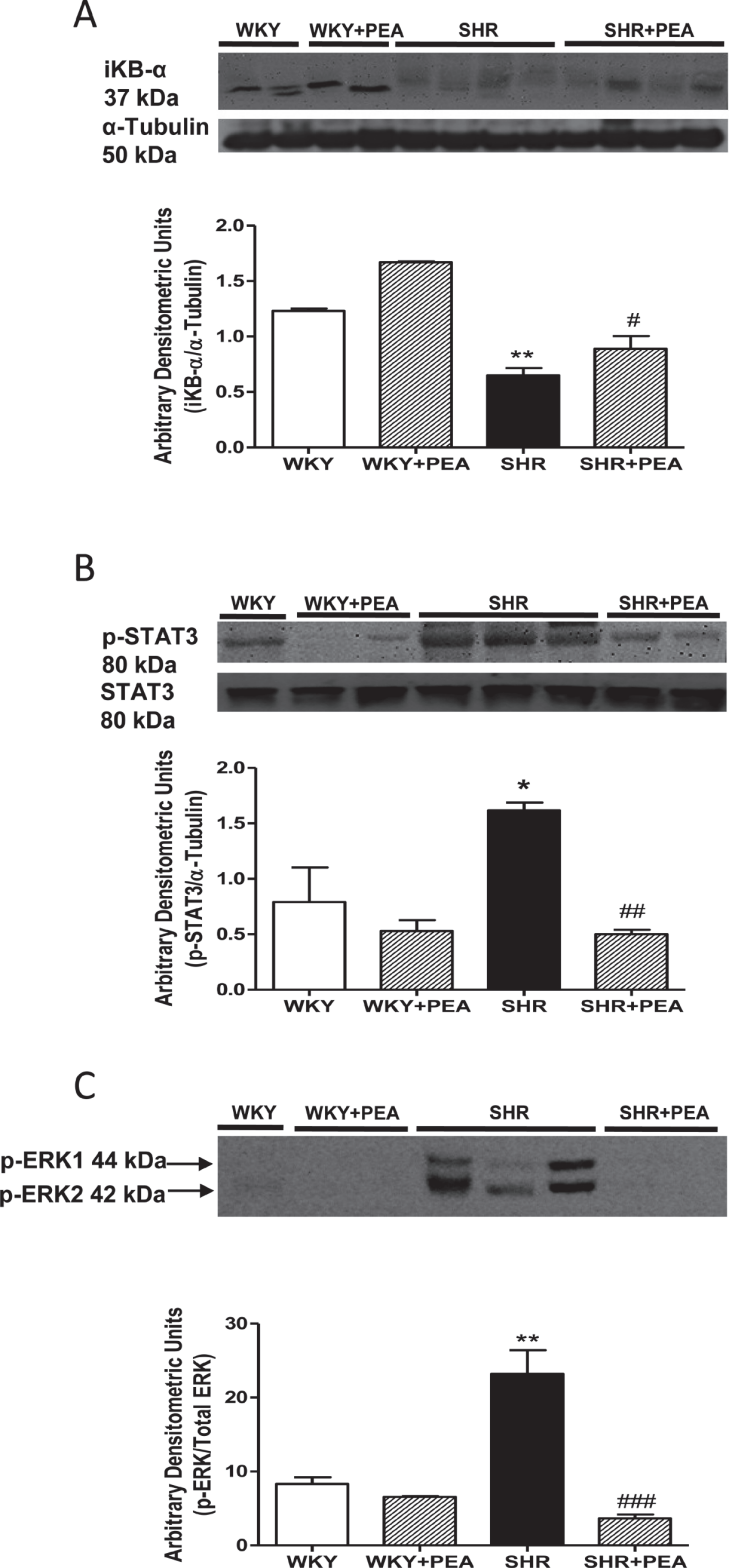
Activation of transcription factors in mesenteric bed and its modulation by PEA treatment. IκBα (A), pSTAT3/STAT (B), and pERK1/2 (C) immunoblots are shown. Densitometric evaluations of protein levels were obtained from 5 different animals. Data are expressed as means ± SEM. *P<0.05 and **P<0.01 vs WKY; #P<0.05, ##P<0.01 and ###P<0.001 vs SHR.

To evaluate the modulation of AT1 activation following PEA treatment, we analyzed several signaling pathway downstream this receptor, such as nuclear factor-κB (NF-κB) activation through IκBα degradation, and STAT3 and ERK1/2 phosphorylated state. Indeed, sEH is a novel targeting gene regulated by Ang II, in fact one NF-κB binding site and three AP-1 putative binding sites were found in sEH promoter, responsible of sEH induction by Ang II [11,15]. As shown in Fig 5, PEA treatment partially prevented IκBα degradation (Fig 5A), reduced the increased phosphorylation of STAT3 (Fig 5B) and ERK1/2 (Fig 5C) in SHRs, attenuating the increase of AT1-mediated signaling in SHR basal conditions.

## Discussion

In the present study, we show that long-term treatment of SHR with PEA alleviates hypertension, by improving EDHF-mediated vasodilation, through the modulation of EET hydrolisis and renin-angiotensin system in the vasculature. Our major finding, besides PEA-induced reduction of blood pressure, was the capability of PEA in EDHF-mediated improvement of vasodilator function by acetylcholine in pre-constricted mesenteric bed from SHRs. To date, EDHF-like activity can be measured only by bioassay. Indeed, EDHF effect is identified by stimulating resistance vessels with Ach in presence of NOS and COX inhibitors. Here, excluding the contribution of NO and prostaglandins, through L-NAME and INDO treatment, we highlighted the EDHF contribution in vasodilatation of mesenteric bed. Interestingly the EDHF impairment observed in SHR was restored by the treatment with PEA. Interestingly, PEA treatment in SHR caused a significant improvement not only in EDHF, but also in the endothelial dysfunction (as NO and prostacyclin release). In fact, evaluating the tone achieved by adding INDO plus L-NAME, the contraction was significantly higher in SHR treated with PEA compared with SHR, implying a major contribution of endothelial-derived relaxing factors in PEA-treated SHRs. According with our results, recently a clinical study on ocular hypertensive patients showed a reduction in the intraocular pressure following three months PEA treatment, improving the endothelium-dependent flow-mediated vasodilation compared to placebo [21].

Notably, in small resistance arteries, EDHF plays a major role in the response to vasoactive substances and regulation of vascular tone. In particular, the mesenteric vascular bed produces vascular resistance to develop blood pressure and regulate tissue blood flow, playing an important role in maintenance of systemic blood pressure. Basically, whereas in the large conduit vessels, such as the aorta, endothelium-dependent responses are selectively mediated by NO, EDHF is the predominant endothelium-dependent vasodilator in resistance vessels [4, 22].Therefore, the role of EDHF in the resistance arteries is important in cardiovascular diseases, such as hypertension, diabetes mellitus and congestive heart failure [23,24].

The mechanisms for endothelium dysfunction vary depending on the type of blood vessel/vascular bed, and are related to the nature of hypertension. In particular, SHR experimental model is characterized by a reduction of EET plasma levels, a reduction of blood pressure in response to sEH inhibition and an increased expression and activity of sEH compared to normotensive animals [25–27]. Consistently, we found an impairment of EDHF vasodilation elicited by acetylcholine in hypertensive rats. This finding agrees with those reported for mesenteric arteries of the spontaneously hypertensive rat [28] and in salt-sensitive hypertensive patients [29]. Taken together, all these findings prompted us to speculate an involvement of EET/DHET metabolites, as EDHF, in blood pressure lowering effect of PEA, and we found a significant decrease in sEH in vascular tissues in SHR after PEA treatment. Indeed, it was already proposed that increased expression or activity of CYP was found during the development of hypertension in SHR model [13], and interpreted as a compensatory response to the elevation of blood pressure. Consistently, in our study, both vascular tissues, SHR mesenteric bed and carotid artery, showed an increase in CYP2C23 and CYP2J2, reverted in mesenteric bed and unaffected in carotid artery by PEA. Our conceivable conclusion has been, then, a longer half-life of vasodilating EETs in the blood as a result of their reduced catabolism to the corresponding DHETs. We confirm this hypothesis, showing a significant reduction in sEH expression in the vasculature and in serum 14,15-DHET in PEA-treated SHR, demonstrating, indirectly, an increase in vascular-derived EETs levels. The central role of sEH in the initiation and establishment of hypertension has been confirmed by evidence showing that treatment with sEH-selective inhibitors in Ang II-infused hypertensive rats increases the level of EETs, with attendant decrease in systolic blood pressure [14].

In the large, elastic, superior mesenteric artery of aged SHRs, EDHF-mediated hyperpolarization and relaxation are severely attenuated, but completely restored and even augmented after inhibition of the RAS [30,31]. Thus, we also focused on this pathway: in our study, RAS modulation is in agreement with previous findings, since SHRs showed an increase in AT1 and ACE expression, that was prevented by PEA treatment. Consistently with our findings, clinical evidence have demonstrated that the actions of Ang II extend far beyond classical actions of RAS, but are also linked to wide array of cellular pathways including that of inflammatory processes, as well as to endothelial disorders with reduction in endothelium-derived relaxing factors [12]. However, in rats, ACE has been shown to be the most important enzyme for Ang II formation [32]. Evidence indicate that several tissues, including vasculature, contain all components of the RAS and are thus capable of producing local Ang II [33]. Interestingly, vascular inflammatory response has been shown to be more closely related to local than circulating Ang II [34]. Therefore, local Ang II appears to be more important in the regulation of Ang II-induced inflammation.

An interaction between Ang II and sEH has been established, in fact, the level of this enzyme in the heart and endothelium is upregulated by Ang II *in vitro* in cultured cardiomyocytes and vascular endothelial cells and *in vivo* in rodent models, leading to a reduction of half life of vasodilating and anti-inflammatory EETs [11]. Therefore, the attenuation of RAS activity, shown by AT1 and ACE down-regulation observed in PEA treated rats, could be responsible, at least in part, to the reduced expression of sEH found in PEA-treated SHRs. Consistently, we showed an attenuation of key downstream factors of Ang II signaling cascade; in particular, PEA treatment increased IκBα content in SHR, demonstrating a reduction of NF-κB activation, and a reduction of the phosphorylated state of STAT3 and ERK1/2. The damping of AT1-mediated signaling cascade by PEA indicates the reduction of transcription factor activation, leading to the modulation of downstream genes, including sEH transcription.

We conclude that PEA treatment alleviates hypertension, improving EDHF-mediated vasodilation of mesenteric arteries. This effect is related to the increase in EET half-life, due to a decrease in the expression of sEH, the hydrolyzing enzyme. On the other side, PEA treatment reduces AT1 expression and hence Ang II-mediated effects, indicating a further mechanism contributing to its blood pressure lowering effect. Therefore, PEA could modulate in a concerted way the interaction among Ang II and sEH/EET system, being useful in reinforcing anti-hypertensive therapy. Currently, ACE inhibitors and AT1-receptor blockers, as RAS interfering agents, are widely used in anti-hypertensive treatment. However, a part from their troublesome side effects, these compounds do not completely inhibit RAS, as a result of Ang II formation by indirect pathways and compensatory feedback mechanisms, resulting in renin release and AT1 over-expression [35]. Thus, PEA, reducing AT1 and ACE expression the major targets of anti-hypertensive therapy, as well as of sEH, may be considered a supplemental approach to blockade of RAS. This state-of-affairs supports the notion that a combined therapeutic strategy of the aforementioned anti-hypertensive drugs and PEA would be more efficacious, since the ACE inhibition or AT1 blocking, would be strengthened through the reduced expression of these drug targets by PEA.
